# EEG Microstates Are Associated with Motor Function in Parkinson's Disease: A Cross‐Sectional Observational Study

**DOI:** 10.1002/brb3.71151

**Published:** 2026-01-13

**Authors:** Jiayu Cai, Yuqing Zhao, Jian Song, Xianling Xu, Jinfeng Xu, Haoran Shi, Wei Wei, Xiehua Xue

**Affiliations:** ^1^ The Affiliated Rehabilitation Hospital Fujian University of Traditional Chinese Medicine Fuzhou China; ^2^ College of Rehabilitation Medicine Fujian University of Traditional Chinese Medicine Fuzhou China; ^3^ Fujian Provincial Key Laboratory of Rehabilitation Technology, Fujian Key Laboratory of Cognitive Rehabilitation Fujian Provincial Rehabilitation Industrial Institution Fuzhou China

**Keywords:** Parkinson's disease, motor symptoms, microstate analysis, EEG

## Abstract

**Objectives:**

Electroencephalogram (EEG) microstates can precisely capture transient brain activity changes on sub‐second time scales and are used to evaluate global dynamic functional alterations in the brains of Parkinson's disease (PD) patients. This study primarily investigates the differences in microstate features between PD patients and healthy subjects, while exploring their correlations with clinical symptoms.

**Methods:**

We enrolled 75 PD patients and 44 healthy controls (HCs) who underwent simultaneous EEG microstate recording and Montreal Cognitive Assessment (MoCA) evaluation. All PD patients underwent comprehensive evaluation using the International Movement Disorder Society Unified Parkinson's Disease Rating Scale Parts I and III (MDS‐UPDRS I & III).

**Results:**

PD patients demonstrated significantly increased mean coverage and duration of microstate D compared with HCs, together with elevated transition probabilities from microstates A, B, and C to D. Importantly, both the mean coverage of microstate D and the transition probability from microstate B to D showed significant positive correlations with MDS‐UPDRS III scores. Furthermore, receiver operating characteristic (ROC) curve analysis revealed that the mean coverage of microstate D (AUC = 0.674) and the transition probability from microstate B to D (AUC = 0.617) could distinguish between PD patients and HCs.

**Conclusion:**

The observed abnormalities in microstate dynamics among PD patients may stem from an imbalance in neural network dynamics. These results indicate that EEG microstates may serve as potential biomarkers for assessing and monitoring motor function in PD.

## Graphical abstract

Parkinson's disease patients exhibited distinct abnormalities in microstate D, including increased mean coverage, mean duration, and incoming transition probabilities. Notably, both the coverage of microstate D and the transition probability from microstate B to D correlated with motor symptom severity, suggesting their potential as biomarkers for assessing motor function.

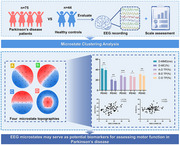

## 1 Introduction

Parkinson's disease (PD), a prevalent neurodegenerative disorder, currently affects an estimated six million people globally, with rising incidence tied to aging populations (GBD 2016 Neurology Collaborators 2019). The core pathological hallmark of the disorder is characterized by progressive degeneration of dopaminergic neurons in the substantia nigra (Armstrong and Okun 2020). This multisystem condition presents with diverse clinical features ranging from characteristic motor impairments (e.g., bradykinesia, resting tremor, and gait abnormalities) to various non‐motor manifestations (e.g., cognitive decline and mood disturbances; Hurben and Tretyakova 2022). With the advancement of the disease, these symptoms are aggravated, which significantly impacts the living standards of both patients and their families (Ou et al. 2021).

Current clinical assessment of PD faces persistent challenges, primarily relying on subjective rating scales that may inadequately capture the multidimensional complexity of the disease. To achieve accurate assessments, it is critical to establish standardized objective measures with demonstrated reliability.

Recent studies have indicated that dynamic alterations in brain activity have been identified as a pivotal component in the pathogenic mechanism of PD (Serrano et al. 2018). As a non‐invasive assessment tool, the electroencephalogram (EEG) captures both spatial distributions and temporal fluctuations of bioelectrical brain activity (Schumacher et al. 2019, Acharya et al. 2018). EEG microstate analysis surpasses functional magnetic resonance imaging (fMRI) in temporal precision, providing sub‐second timescales tracking of rapid brain dynamics (Chu et al. 2020). Researchers define EEG microstates as global scalp potential topography patterns from multichannel EEG systems that show organized dynamic transitions temporally (Michel and Koenig 2018). It is noteworthy that numerous prior research has documented the four canonical EEG microstates (A–D), each demonstrating topographic stability lasting 80–120 ms before transitioning to another state (Khanna et al. 2015).

It has been shown that EEG microstates reflecting abnormal brain dynamics can serve as quantifiable markers for various neurological disorders and correlate with clinical predictors (Chu et al. 2020, Smailovic et al. 2019, Soni et al. 2019, Gschwind et al. 2016). Chu et al. (2020) elucidated cerebral dynamics abnormalities in early‐onset, unmedicated PD patients and established a correlation with treatment. EEG microstate analysis identified a decreased incidence of microstate D in the PD population, supporting its potential as a biomarker (Pal et al. 2021). Consequently, EEG microstates may serve as valuable adjuncts in PD assessment, providing insights into the functional brain activity of affected individuals.

Furthermore, PD demonstrates widespread abnormalities in key functional networks, primarily involving the default mode network, salience network, frontoparietal network, and sensorimotor/visual network (Putcha et al. 2015, Tinaz et al. 2016, Baggio et al. 2014, Li et al. 2021). Each type of microstate has been associated with specific resting‐state networks (Britz et al. 2010, Yuan et al. 2016), whose dynamic interactions are implicated in PD pathophysiology (Khanna et al. 2015).

However, the characteristics of EEG microstates in PD patients and their associations with clinical manifestations remain unclear. This study primarily investigates the differences in microstate features between PD patients and healthy subjects, while exploring their correlations with clinical symptoms. The aim is to identify reliable quantitative biomarkers for assessing PD.

## 2 Method

### 2.1 Participants

Seventy‐five PD patients and forty‐four healthy controls (HCs) were recruited from March 2024 to April 2025. PD patients met the international Clinical Diagnostic Criteria for Parkinson's Disease MDS (2015 edition), aged between 45 and 80 years without significant head tremor, and were capable of completing neuropsychological assessments and resting‐state EEG recordings. Exclusion criteria comprised: (1) patients diagnosed with parkinson's syndrome or any superimposed syndromes; (2) individuals with serious medical conditions or complications, including stroke or tumors; (3) patients with depression, schizophrenia, or other neurological degeneration; (4) persons with primary or secondary epilepsy, severe visual, hearing, and speech dysfunction. HCs aged 45–80 years with no neurological or psychiatric history were recruited in parallel. Patients with PD underwent all assessments during the ON medication state. All participants and their family members reviewed the study protocol and completed written consent agreements. All procedures adhered to the Declaration of Helsinki and received formal approval from the Ethics Committee of the Rehabilitation Hospital Affiliated to Fujian University of Traditional Chinese Medicine (No. 2023KY‐056‐002).

### 2.2 Data Collection and Scale Assessment

#### 2.2.1 General Demographic Information

General demographic information was collected from the participants, including age, gender, Hoehn‐Yahr (H&Y) stage, educational level, levodopa equivalent daily dose (LEDD), disease duration, hypertension, and diabetes.

#### 2.2.2 Clinical Scale Assessment

The PD group utilized the Movement Disorder Society Unified Parkinson's Disease Rating Scale Part III (MDS‐UPDRS III) to evaluate motor function (Goetz et al. 2008). Non‐motor symptoms were assessed using the MDS‐UPDRS Part I (MDS‐UPDRS I; Goetz et al. 2008). Cognitive function was evaluated using the Montreal Cognitive Assessment (MoCA) scale (Dalrymple‐Alford et al. 2010).

### 2.3 EEG Acquisition and Microstate Analysis

Participants in this study completed resting‐state EEG recordings while maintaining eyes‐closed, quiet wakefulness. EEG data acquisition was conducted in compliance with the International 10–20 System standards, employing the specialized cognitive‐autonomic assessment apparatus (NVX52 EEG System, Nanjing NeuroMed Technology Group Co., Ltd, China). The system employed 19 AgCl electrodes fixed in position using an adjustable electrode cap, with a computed reference electrode (AA) derived from the averaged signals of A1 and A2. The EEG acquisition time for each subject was 3 min. Continuous EEG was recorded at 500 Hz sampling frequency with all electrode impedances maintained below 30 kΩ using the average reference montage. The raw EEG signals were digitally filtered (finite impulse response band‐pass: 1–35 Hz) to remove artifacts (e.g., low‐frequency drifts, high‐frequency noise). The EEG signal was decomposed using independent component analysis (ICA) to identify and subsequently remove artifacts associated with blinks, muscle contractions, and eye movements. Following the processing of artifacts, a 60 s segment was used for analysis for each participant.

**FIGURE 1:**
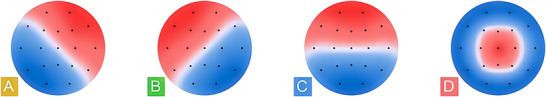
Topographic maps of four microstates.

EEG microstates were extracted from the raw data following the methodology described by Musaeus et al. (2020). The global field power (GFP) was computed as a metric for the instantaneous signal strength of the brain's electric field. Due to their possession of a high signal‐to‐noise ratio and exhibition of stable peripheral topographic maps, GFP peaks were chosen for the ensuing clustering analysis (Grave de Peralta Menendez et al. 2004). The formula for GFP is represented as Equation (1):

(1) $$\mathrm{GFP}\left(t\right)=\sqrt{\frac{\sum_{i=1}^{N}{\left(V_{i}\left(t\right)-\bar{V}\left(t\right)\right)}^{2}}{N}},$$

where *N* is the total number of electrodes; $V_{i}(t)$ represents the recorded electric potential at the i‐th electrode at the time point t; $\bar{V}\left(t\right)$ represents the average electric potential across all N electrodes at the time point t. The minimum peak spacing was set to 10 ms to distinguish neighboring peaks better. GFP peaks beyond two standard deviations from the mean GFP of all maps were removed. Both K‐means clustering and cross‐validation studies have consistently established four as the optimal number of clusters in subjects (Britz et al. 2010, Brodbeck et al. 2012). Accordingly, the four microstate classes were predefined (A: right–left asymmetry; B: left–right asymmetry; C: horizontal symmetry; D: circular symmetry; see Figure 1).

Microstate topographic clustering was performed using a dynamic initialization approach. Representative candidate maps were initially identified by screening topographies at GFP peaks, which yielded a variable number of candidate maps for potential microstate classes. A comprehensive grid search was subsequently performed by evaluating all possible combinations of these candidate map sets. For each combination, the clustering algorithm was executed with a maximum of 500 iterations and a convergence threshold of 10^−6^. The optimal microstate set was determined by selecting the configuration that maximized the global explained variance (GEV). To eliminate the influence of noisy transient segments, microstate segments with a duration of less than 8 ms were excluded. Following template selection, a back‐fitting procedure was implemented to characterize the temporal dynamics of these microstates in continuous EEG data. Each time point in the preprocessed EEG was assigned to the microstate template demonstrating the highest absolute spatial correlation, thereby generating a continuous sequence of microstate labels for each participant that represents the moment‐to‐moment dominance of specific brain networks. Finally, the processing pipeline generated these derived microstate indices: mean microstate duration (MMD), mean frequency occurrence (MFO), mean coverage (MC), and transition probability (TP). The transition probabilities between microstates were calculated by applying row‐wise normalization to the raw count matrix to eliminate the influence of differences in the total number of microstate occurrences. Specifically, the counts in each row of the matrix (representing all transitions from a given predecessor state) were normalized by division by the row sum, such that the values in every row summed to 100%.

### 2.4 Statistical Analysis

Continuous variables following normal distributions were expressed as mean ± standard deviation (SD), and group comparisons were conducted using independent Student's *t*‐tests. Nonnormally distributed data were analyzed with the Mann–Whitney *U* test, reported as median (interquartile range [IQR]). Categorical variables were compared using either Fisher's exact test or Pearson's chi‐square test, as appropriate for the sample characteristics. Pearson's correlation analysis was employed when both variables met the assumption of normal distribution, while Spearman's correlation analysis was utilized when at least one variable did not meet this assumption or was ordinal in nature. To account for multiple testing in correlation analyses, false discovery rate (FDR) correction was implemented. For group discrimination between PD and HC subjects, we evaluated the diagnostic performance using receiver operating characteristic (ROC) analysis. The ROC curve was constructed by plotting the true positive rate (TPR = Sensitivity = TP/(TP+FN)) against the false positive rate (FPR = 1 − Specificity = FP/(FP+TN)) across a continuum of discrimination thresholds. The area under the curve (AUC) was computed to evaluate the overall discriminative power. The AUC value was calculated using the trapezoidal rule method, with the formula given in Equation (2):

(2) $$\mathrm{AUC}=\sum_{i=1}^{n}\frac{(\mathrm{FP}R_{i}-\mathrm{FP}R_{i-1})\times (\mathrm{TP}R_{i}+\mathrm{TP}R_{i-1})}{2},$$

The optimal cut‐off value was identified as the threshold that maximized Youden's index (*j*), defined as *j* = Sensitivity + Specificity − 1. Statistical significance was defined as *p *< 0.05 in this investigation.

## 3 Results

### 3.1 General Demographic Data

No significant intergroup differences were observed in demographic characteristics, including age, gender distribution, educational level, hypertension, and diabetes (*p *> 0.05). For PD patients, the disease duration was [3.00(2.00,5.00)] years, the LEDD was [0.38(0.30,0.53)] grams, and the H&Y stage of [2.00(2.00,2.00)], as described in Table 1. We assessed the potential influence of LEDD on microstate outcomes. Correlation analyses revealed no significant associations between LEDD and any of the significant microstate parameters (*p *> 0.05), see Figure S1. Furthermore, dividing PD patients into high‐ and low‐LEDD subgroups based on the median dose showed no statistically significant differences in any microstate parameters between them (*p *> 0.05), see Table S1. Consistently, these findings indicate that LEDD is unlikely to be a major confounding factor in the EEG microstate differences observed in this study.

**TABLE 1:** Comparison of baseline data between the PD and HC groups.

	PD group (*n* = 75)	HC group (*n* = 44)	*x^2^/Z*	*p‐*value
Age (years)	66.00±7.83	69.00(64.25,73.00)	−1.701	0.089
Gender(F/M)	41/34	23/21	0.064	0.800
Educational level (years)	12.00(6.00,15.00)	12.00(9.00,16.00)	−1.604	0.109
Hypertension (Y/N)	18/57	8/36	0.550	0.458
Diabetes (Y/N)	3/72	3/41	0.060	0.807
H&Y stage	2.00(2.00,2.00)	—	—	—
LEDD (g)	0.38(0.30,0.53)	—	—	—
Disease duration (years)	3.00(2.00,5.00)			
MDS‐UPDRS I	6.00(3.00,10.00)	—	—	—
MDS‐UPDRS III	31.00±12.76	—	—	—
MoCA‐total	22.00(17.00,26.00)	26.00(26.00,26.00)	−5.626	<0.001^a^

^a^Significant differences between groups.

Abbreviations: F: Female; H&Y stage: Hoehn‐Yahr stage; HC: Healthy control; LEDD: Levodopa equivalent daily dose; M: Male; MDS‐UPDRS I: MDS‐Unified Parkinson's Disease Rating Scale, Part I; MDS‐UPDRS III: MDS‐Unified Parkinson's Disease Rating Scale, Part III; MoCA: Montreal Cognitive Assessment; N: No; PD: Parkinson's disease; Y: Yes.

### 3.2 Comparison of Clinical Scales Between Two Groups

PD patients scored [6.00(3.00, 10.00)] points in the MDS‐UPDRS I and 31.00 ± 12.76 points in the MDS—UPDRS III. A statistically significant difference in MoCA total scores was observed between the two groups (*Z* = −5.626, *p* < 0.001), as described in Table 1.

### 3.3 Comparison of the MMD, MFO, and MC Between Two Groups

**FIGURE 2:**
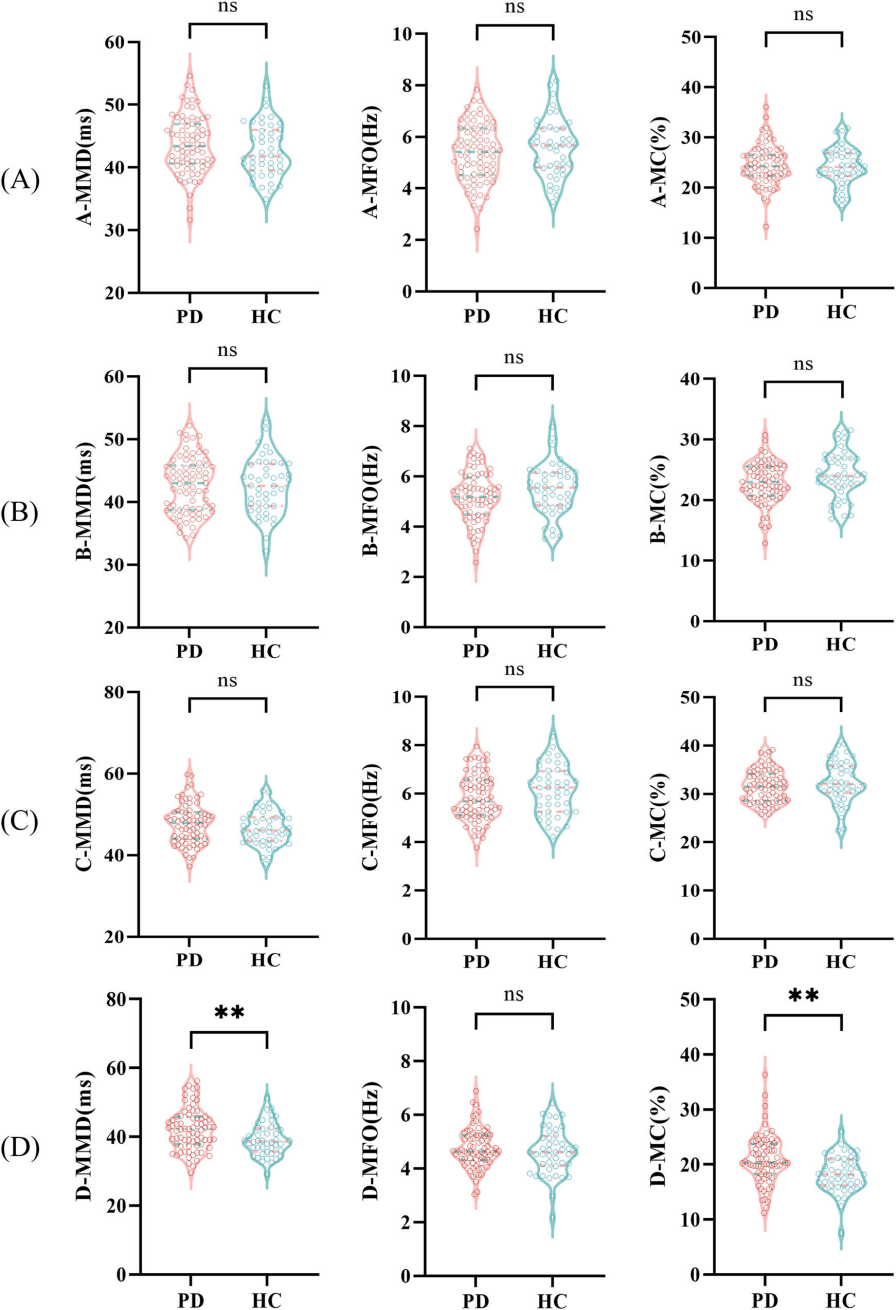
Comparison of the MC, MFO, and MMD between the PD and HC groups. (A) Comparison of MMD, MFO, and MC for microstate A between the PD and HC groups. (B) Comparison of MMD, MFO, and MC for microstate B between the PD and HC groups. (C) Comparison of MMD, MFO, and MC for microstate C between the PD and HC groups. (D) Comparison of MMD, MFO, and MC for microstate D between the PD and HC groups. Significant differences between groups are indicated by *. **p < 0.01, ^ns^ *p *> 0.05.

A significant between‐group difference was observed in both the MMD (*t* = 2.831, *p* = 0.005) and MC (*Z* = −3.432, *p* = 0.001) of microstate D when comparing PD patients with HCs, while no differences were found in other variables (*p *> 0.05), as shown in Figure 2. GEV measurements revealed no significant differences between the two groups (*p* > 0.05)

### 3.4 Comparison of the Transition Probabilities of Microstates Between Two Groups

**FIGURE 3:**
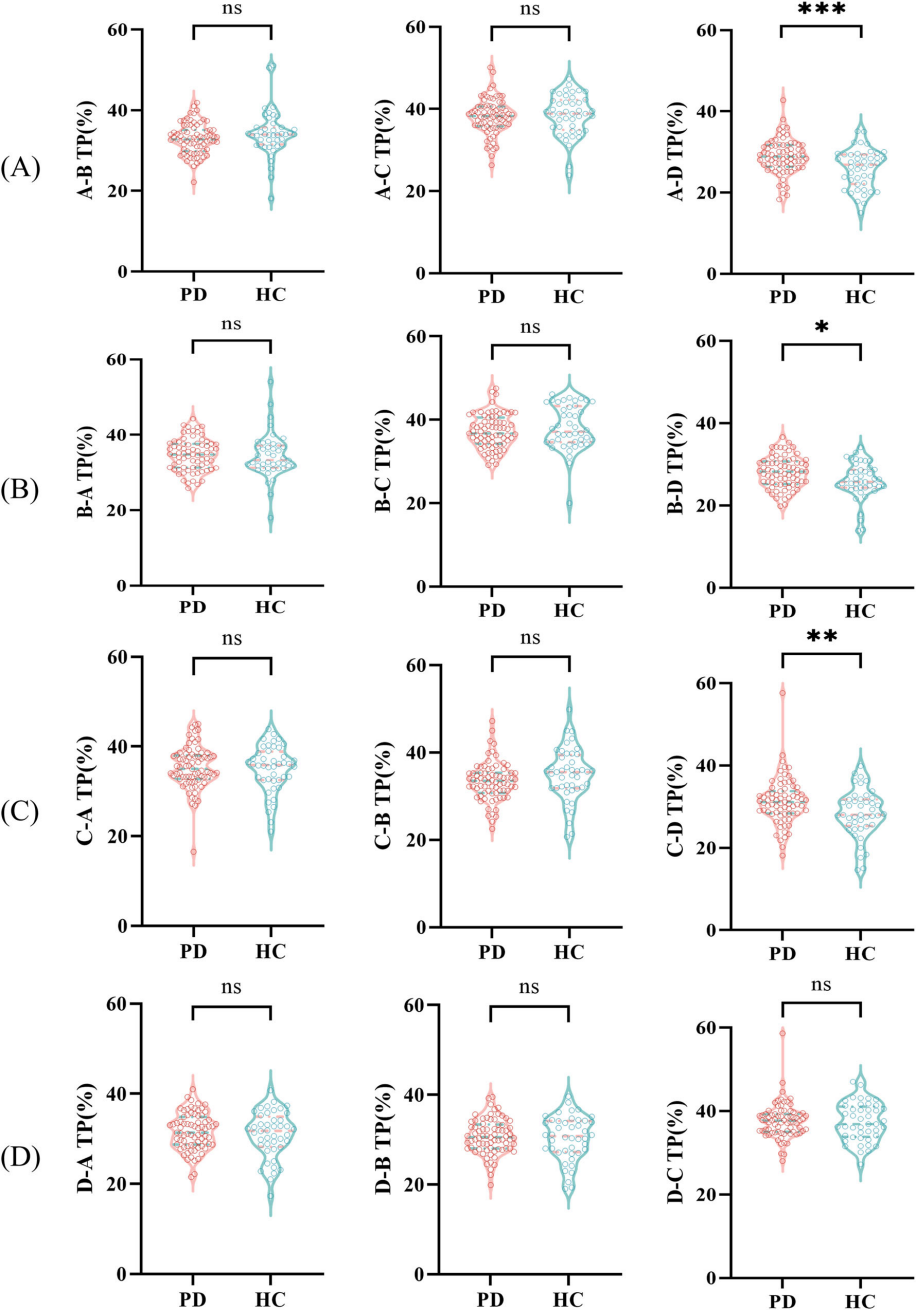
Comparison of transition probabilities of microstates between the PD and HC groups. (A) The comparison of transition probabilities from microstate A,B, A‐C, A‐D between PD and HC groups. (B) The comparison of transition probabilities from microstate B‐A, B‐C, B‐D between PD and HC groups. (C) The comparison of transition probabilities from microstate C‐A, C‐B, C‐D between PD and HC groups. (D) The comparison of transition probabilities from microstate D‐A, D‐B, D‐C between PD and HC groups. Significant differences between groups are indicated by *. *p < 0.05, **p < 0.01, ^ns^ *p *> 0.05.

Compared with HCs, the transition probabilities from microstate A to D (*t* = 3.612, *p* < 0.001), B to D (*Z* = −2.378, *p* = 0.017), and C to D (*Z* = −2.659, *p* = 0.008) were found to be significantly higher in the PD group. The rest of the indices exhibited no statistically significant differences (*p *> 0.05), as shown in Figure 3.

### 3.5 Correlation Analysis of Microstate Parameters with Clinical Scales

**FIGURE 4:**
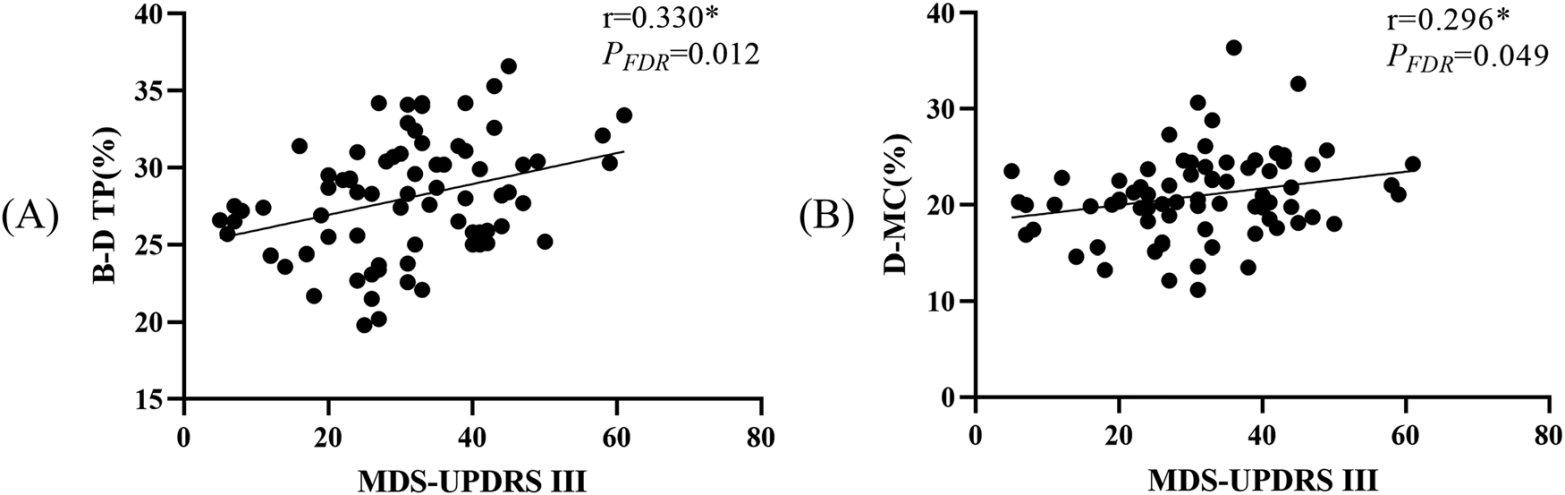
Correlation analysis of microstate parameters with MDS‐UPDRS III. (A) The correlation between MDS‐UPDRS III and the transition probability of microstate B‐D. (B) The correlation between MDS‐UPDRS III and mean coverage of microstate D. Significant differences between groups are indicated by *.

Statistical analyses demonstrated significant positive associations between MDS‐UPDRS III scores and specific microstate dynamics in PD patients, particularly the transition probability from microstate B to D (*r* = 0.330, *P*_FDR_ = 0.020) and the MC of microstate D (*r* = 0.269, *P*_FDR_ = 0.049). However, neither MDS‐UPDRS I scores nor MoCA scores showed any significant correlation with microstate parameters (*p *> 0.05), as shown in Figure 4.

### 3.6 Analysis of the ROC Curve

**FIGURE 5:**
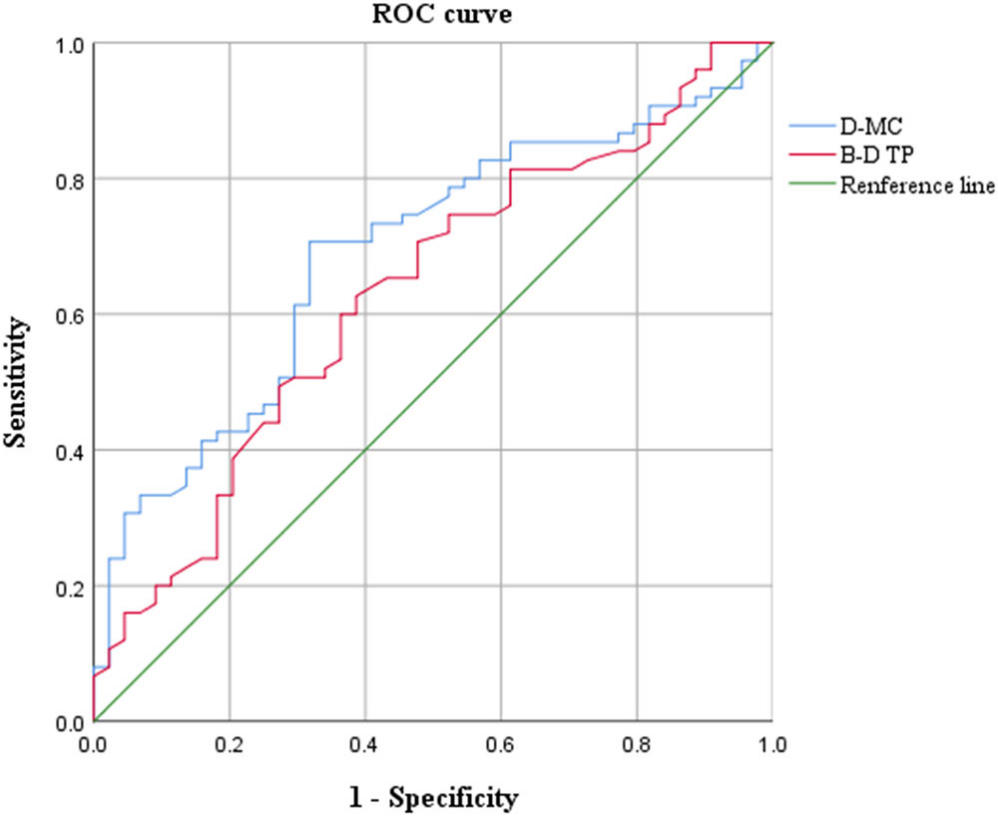
ROC analysis was used to estimate the microstate parameters for distinguishing between PD patients and HCs.

We selected the transition probability from microstate B to D and the MC of microstate D for ROC analysis due to their demonstrated correlations with motor function. To assess the discriminative ability of transition probability from microstate B to D and MC of microstate D in differentiating PD patients from HCs, we performed ROC curve analyses. The AUC of transition probability from microstate B to D was 0.617 (95% CI: 0.512–0.721, *p* = 0.032), with a sensitivity of 62.7%, a specificity of 61.4%, and a cut‐off value of 26.55%. For MC of microstate D, the AUC was 0.674 (95% CI: 0.575–0.772, *p* = 0.001), with a sensitivity of 70.7%, a specificity of 68.2%, and a cut‐off value of 19.66%. These findings demonstrate that both metrics could function as potential biomarkers for differentiating PD patients from healthy individuals, as shown in Figure 5.

## 4 Discussion

EEG microstates possess the potential to characterize brain variability and exhibit complex dynamic properties essential for optimal brain function (Schumacher et al. 2019, Li et al. 2021). The findings of this study demonstrated that patients with PD exhibited higher transition probabilities from microstates A, B, and C to D, as well as greater MC and longer MMD of microstate D. Moreover, the severity of motor symptoms in PD patients showed statistically significant positive correlations with both the transition probability from microstate B to D and the MC of microstate D in PD patients.

Microstate D abnormalities (increased MMD/MFO/MC) correlated with attentional dysfunction in PD with mild cognitive impairment (Liu et al. 2023, Zarkali et al. 2021). As microstate D was linked to reflexive attention (Tarailis et al. 2024), its elevation aligns with reported enhancements of reflexive attentional processes in PD versus HCs (Briand et al. 2001). Notably, attentional anomalies correlate with motor deficits in PD (Amboni et al. 2013, Montero‐Odasso and Speechley 2018). Our findings further support this association by demonstrating significantly elevated MC and MMD of microstate D in PD patients, with MC showing significant correlation with MDS‐UPDRS III scores. These results were consistent with the hypothesis that attentional impairments may contribute to motor dysfunction.

Extensive studies have demonstrated that patients with PD exhibited significant abnormalities in brain network organization and functional imbalance, manifested as impaired functional coordination across distributed brain regions (Olde Dubbelink et al. 2014, Oswal et al. 2016, Kim et al. 2017, Ma et al. 2017). Microstate A exhibits a correlation with the auditory network, which is primarily reflected in its strong functional connectivity with the bilateral superior/middle temporal gyrus (Gschwind et al. 2016, Britz et al. 2010). In contrast, Microstate D exhibits strong functional connectivity with the dorsal attention network (Gschwind et al. 2016, Tarailis et al. 2024). These functional networks normally maintain balanced interactions through sensorimotor integration processes. However, research suggests that sensory deficits in PD may stem from disrupted network coordination that exacerbates both sensory and motor symptoms (Degardin et al. 2009, Macerollo et al. 2016, Caspers et al. 2021). For instance, a retrospective review indicated that patients with PD exhibited functional impairments in the superior temporal auditory cortex and basal ganglia during auditory processing (Permezel et al. 2023). As these regions are core components of the auditory‐attentional integration network, their dysfunction strongly suggested the potential occurrence of disrupted interactive coordination within this network (Permezel et al. 2023). In our research, the PD group exhibited a significantly increased transition probability from microstates A to D. It was reported that the transitions among microstates are regarded as nonrandom (Khanna et al. 2015). Consequently, this aberrant transition pattern likely reflected a direct impairment in the coordination between the auditory and attention networks, suggesting that the resulting cross‐network dysregulation disrupted the efficient integration of sensory information with attentional resources (Göttlich et al. 2013).

The current study revealed that there was a significantly higher transition probability from microstate C to D in the PD group relative to the HC group. PD patients commonly experience deficits in cognitive domains such as working memory, visuospatial ability, and attention (Aarsland et al. 2021). Considering previous evidence that the transition probability from microstate C to D may reflect engagement of the dorsal attentional network during cognitively demanding tasks (Cucca et al. 2021), we hypothesize that this increased transition probability could represent compensatory recruitment of attentional resources in PD patients.

We found that PD subjects revealed increased transition probability from microstate B to D than HCs. Additionally, the transition probability from microstate B to D showed a positive correlation with MDS‐UPDRS III scores, indicating that the dynamic reorganization of functional brain networks may correlate with motor symptom progression. Microstate B is implicated in visuospatial attention and sensory processing (Tarailis et al. 2024), while microstate D exhibits associations with the dorsal attentional network or the frontoparietal network that are particularly crucial for maintaining attentional stability and modulating motor function in PD (Vossel et al. 2014, Maidan et al. 2019). Prior evidence indicates that visual perception and processing impairments in PD significantly correlate with motor dysfunction severity, while persistent metabolic activity is observed in associated brain regions even in clinically asymptomatic stages (Cucca et al. 2021, Jiang et al. 2016). In the healthy brain, subcortical networks centered on the basal ganglia efficiently perform automatic gating and selection of sensory information, thereby minimizing reliance on higher‐order attentional networks (Esposito et al. 2021, Hikosaka et al. 2019). However, reduced efficiency and increased noise in the posterior striatum and its circuits impair the foundational automatic sensory gating mechanism in PD patients, thus disrupting the dynamic balance between subcortical automatic and cortical controlled processing networks (Esposito et al. 2021, Tommasi et al. 2015, Lee et al. 2010). To compensatorily maintain basic visuospatial processing efficiency, the brain was forced to increasingly recruit attentional networks to perform tasks that would otherwise be handled automatically by subcortical circuits (Esposito et al. 2021). We hypothesize that such compensatory overactivation may inadvertently exacerbate the severity of motor dysfunction (Esposito et al. 2021, Li et al. 2021). The aberrant B–D microstate transition pattern observed in this study may thus reflect a dynamic electrophysiological signature of this dysfunctional, compensatory reorganization of neural networks.

In this study, we found no correlation between MoCA scores and microstate parameters. Unlike previous studies (Chu et al. 2020), our findings may differ partly because all evaluations were performed while patients were in the ON medication state. Additionally, as a global cognitive screening instrument, the MoCA may have limited sensitivity to changes in specific cognitive domains.

While providing novel insights, this research has some limitations. First, the study did not conduct long‐term follow‐up investigations to explore changes in electroencephalographic microstates during disease progression. Second, although PD patients exhibit abnormal brain dynamics compared with HCs, these changes may represent nonspecific pathological alterations. Finally, an important consideration is the potential influence of dopaminergic medications. Despite our analysis revealing no significant correlation of the differential microstate parameters with LEDD and their stability across dosage ranges, we cannot entirely exclude a medication effect. Therefore, future studies should utilize larger sample sizes in both cross‐sectional and longitudinal research, with comparisons to other neurological disorders, to further investigate the application and progression of EEG microstates in the clinical assessment of PD. Moreover, the implementation of an “on–off” medication paradigm in future studies is critical to definitively disentangle disease‐specific effects from those induced by medication.

Furthermore, while this investigation focused exclusively on EEG microstate dynamics in PD, prior studies have validated both EEG spectral features (Liu et al. 2023) and fMRI‐based connectivity measures (Tessitore et al. 2019) as robust biomarkers for PD detection and differential diagnosis. Future studies should integrate multimodal neuroelectrophysiological indices to enhance the accuracy of PD assessment and diagnosis. This approach could also facilitate more comprehensive assessments and analyses of PD patients across different disease stages and subtypes.

## 5 Conclusion

In summary, EEG microstates offer subsecond‐resolution tracking of PD‐related abnormal brain dynamics for motor function assessment. Our study revealed significant alterations in microstate dynamics in PD patients compared with HCs, particularly abnormal increases in MMD and MC of microstate D, together with elevated transition probabilities from microstates A, B, and C to D. Furthermore, we identified significant correlations between specific microstate features and motor performance. These results suggest that the EEG microstate alterations in patients with PD likely reflect an underlying disease process characterized by network dynamic imbalance, which is closely associated with brain dysfunction in these patients. This provides insights into PD pathophysiology and indicates the potential of EEG microstates as biomarkers.

## Author Contributions

Jiayu Cai: conceptualization, methodology, formal analysis, writing – original draft. Yuqing Zhao: investigation, supervision. Jian Song: visualization, writing – review & editing. Xianling Xu: resources, investigation. Jinfeng Xu: data curation, resources. Haoran Shi: investigation, data curation. Wei Wei: supervision, project administration, writing – review & editing. Xiehua Xue: conceptualization, software, funding acquisition, validation.

## Ethics Statement

The studies involving human participants were reviewed and approved by the Local Ethics Committee in the Affiliated Rehabilitation Hospital of Fujian University of Traditional Chinese Medicine in China. The patients/participants provided their written informed consent to participate in this study.

## Funding

This study was supported by the National Key R&D Program of China (No. 2023YFC3503703), Rehabilitation technology innovation center by joint collaboration of ministry of education and Fujian province, Fujian University of Traditional Chinese Medicine (No. X2022005), Open research project of Fujian Key Laboratory of Cognitive Function Rehabilitation (No. XKF2024001, XKF2024003), Rehabilitation of Traditional Chinese Medicine in the High‐level Key Discipline Construction Project of Traditional Chinese Medicine of the State Administration of Traditional Chinese Medicine (No. zyyzdxk‐2023102).

## Conflicts of Interest

The authors declare no conflicts of interest.

## Supporting information




**Supplementary Material**: brb371151‐sup‐0001‐SuppMat.docx

## Data Availability

The data generated during and/or analyzed during the current study are available from the corresponding author on reasonable request.
