# Supplementary material for: EEG Microstates Are Associated with Motor Function in Parkinson's Disease: A Cross‐Sectional Observational Study
Source: Brain Behav. 2026 Jan 13;16(1):e71151. doi: 10.1002/brb3.71151 (PMC12796831; doi:10.1002/brb3.71151)
Supplement: Supplementary file 1 — Supplementary Material: brb371151‐sup‐0001‐SuppMat.docx [file BRB3-16-e71151-s001.docx]

**Supplementary material**


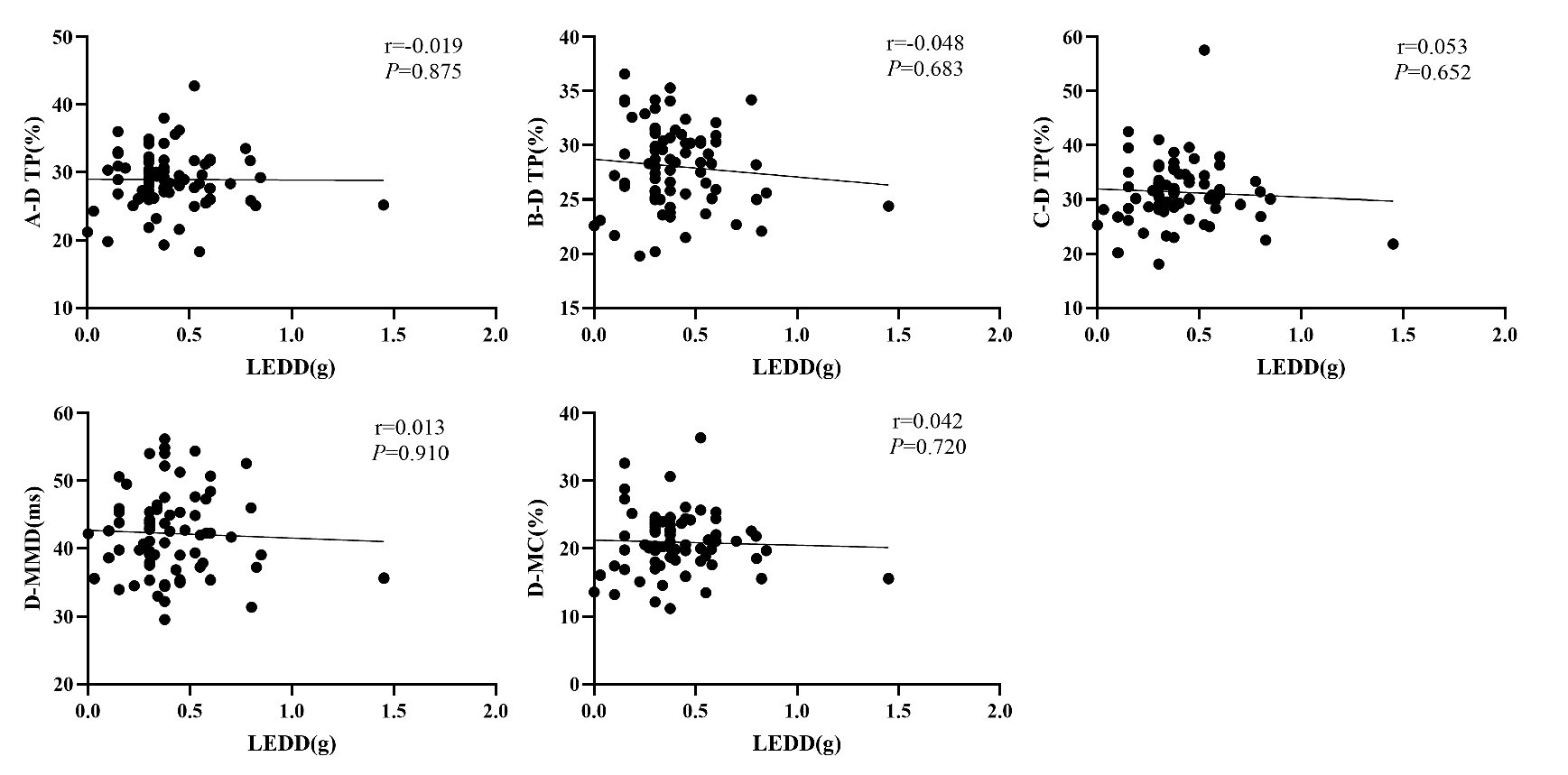


**Supplementary Figure 1.** The correlation between LEDD and microstate parameters.

We calculated the correlations between the levodopa equivalent daily dose and each significantly altered microstate parameter. The results demonstrated no significant correlations (P > 0.05), suggesting that the microstate parameters are not substantially influenced by medication dosage (Supplementary Figure 1).

**Supplementary Table 1.** Comparison of Microstate Parameters between High and Low LEDD Subgroups in Patients with PD

|  | High (n=35) | Low (n=40) | t*/Z* | *P* |
| --- | --- | --- | --- | --- |
| A-MMD (ms) | 43.70±3.89 | 43.63±5.11 | 0.059 | 0.953 |
| A-MFO (Hz) | 5.45±1.16 | 5.32±1.10 | 0.749 | 0.630 |
| A-MC (%) | 24.75±3.80 | 24.38±4.08 | 0.412 | 0.681 |
| B-MMD (ms) | 43.02±4.30 | 42.48±4.67 | 0.517 | 0.607 |
| B-MFO (Hz) | 5.25±8.43 | 5.11±1.10 | 0.634 | 0.528 |
| B-MC (%) | 23.48±2.69 | 22.34±3.80 | 1.479 | 0.143 |
| C-MMD (ms) | 47.52±4.15 | 47.75±5.40 | -0.200 | 0.842 |
| C-MFO (Hz) | 5.77±0.92 | 5.88±1.01 | -0.484 | 0.630 |
| C-MC (%) | 31.28±3.32 | 31.95±3.39 | -0.859 | 0.393 |
| D-MMD (ms) | 41.73±4.70 | 42.67±7.12 | -0.683 | 0.497 |
| D-MFO (Hz) | 4.72±0.65 | 4.78±0.81 | -0.300 | 0.765 |
| D-MC (%) | 20.49±4.44 | 21.33±4.43 | -0.827 | 0.411 |
| A-B TP (%) | 33.63±3.78 | 32.27±3.68 | 1.576 | 0.119 |
| A-C TP (%) | 37.66±4.49 | 38.61±4.13 | -0.960 | 0.340 |
| A-D TP (%) | 28.72±3.87 | 29.11±4.59 | -0.400 | 0.690 |
| B-A TP (%) | 35.36±4.65 | 34.28±3.81 | 1.110 | 0.271 |
| B-C TP (%) | 36.50±4.50 | 37.79±3.60 | -1.371 | 0.174 |
| B-D TP (%) | 28.14±4.18 | 27.97±3.45 | 0.199 | 0.843 |
| C-A TP (%) | 35.91±4.70 | 34.80±4.80 | -0.648 | 0.517 |
| C-B TP (%) | 33.55±4.81 | 33.22±4.08 | 0.328 | 0.744 |
| C-D TP (%) | 30.54±5.18 | 31.99±6.01 | -1.009 | 0.313 |
| D-A TP (%) | 31.58±4.24 | 31.73±3.88 | -0.152 | 0.880 |
| D-B TP (%) | 31.63±3.44 | 29.86±4.05 | -1.652 | 0.099 |
| D-C TP (%) | 36.79±3.57 | 38.42±4.58 | -1.328 | 0.184 |

Subgroup Analysis: To further investigate the effect of medication, we compared microstate parameters between “high-LEDD” and “low-LEDD” PD subgroups, stratified by the median levodopa equivalent daily dose (LEDD). A comparison of microstate parameters between these subgroups revealed no statistically significant differences in any of the parameters (*P*＞0.05). This finding further supports the results of the correlation analysis above, indicating that the microstate patterns remained stable across patients with varying medication doses in this study (Supplementary Table 1).
